# Supplementary material for: Laminin-α2 chain deficiency in skeletal muscle causes dysregulation of multiple cellular mechanisms
Source: Life Sci Alliance. 2024 Oct 8;7(12):e202402829. doi: 10.26508/lsa.202402829 (PMC11463332; doi:10.26508/lsa.202402829)
Supplement: Supplementary file 1 [file LSA-2024-02829_TableS1.docx]

**Supplementary Material**

**Supplementary Table 1.** List of the 191 genes with active expression based on H3K4me3 marker in C2C12 myocytes (i.e. differentiated cells) but downregulated in the RNAseq analysis of muscle fibers (described in Figure 4D).

| Gene Symbol | Gene Symbol | Gene Symbol | Gene Symbol | Gene Symbol |
| --- | --- | --- | --- | --- |
| 1700008O03Rik | Cabp1 | Gm773 | Ncf2 | Slc6a4 |
| 1700017G19Rik | **Cacna1s** | Gm7854 | Nefl | Spats1 |
| 1700061J23Rik | Car5b | Gm960 | Neil1 | Tbx4 |
| 1700112H15Rik | Car6 | Gpr137c | Nkx2-2os | Tcf21 |
| 1700112J16Rik | Casz1 | Gpr156 | Nlrx1 | Tcte2 |
| 2610027K06Rik | Catsperg1 | Grhl1 | Nod2 | Tigd5 |
| 2610035F20Rik | Ccdc172 | Grik5 | Nog | Tmem154 |
| 2900005J15Rik | Ccdc87 | H2-T10 | Nrap | Tmem174 |
| 4632428C04Rik | Ccdc88b | Hapln4 | Nudt15 | **Tmem182** |
| 4921525O09Rik | Cdkl4 | Hey2 | Nyx | Tmem74 |
| 4930405A10Rik | Cdsn | Hid1 | Obscn | Trim54 |
| 4930435F18Rik | Cercam | Hmcn2 | Odf4 | Trp63 |
| 4930449E01Rik | Ces5a | Hsf4 | P2rx3 | Tsks |
| 4930558J18Rik | Chrng | Hunk | Pax6 | Tspan32 |
| 4930565D16Rik | Cntd1 | Ifit1 | Pdzd7 | Ttc16 |
| 4930583K01Rik | Crb2 | Il11 | Phldb3 | Ttll13 |
| 4933406I18Rik | Crppa | Iqank1 | Phtf1os | Usp17la |
| 5430421F17Rik | Cstdc2 | Isl1 | Platr14 | Vinac1 |
| 6430550D23Rik | Dbndd1 | Islr2 | Platr26 | Vmn1r35 |
| 9230102K24Rik | Efcab1 | Kcnip3 | Plpp7 | Wfikkn2 |
| 9330104G04Rik | Efhb | Kcnj12 | Ppfia4 | Xirp1 |
| 9330111N05Rik | Efhd1 | Kif6 | Prdm8 | Zbtb49 |
| 9430014N10Rik | Eomes | Klhdc8b | Proser2 | Zfp493 |
| A830009L08Rik | Esrrb | Klhl14 | Ptpn5 | Zfp536 |
| Acsbg1 | Extl1 | Klhl33 | Rab3c | Zfp764 |
| Agap2 | F2 | Lncenc1 | Reep6 | Zfp831 |
| Alpk2 | Fbxl21 | Lrrc61 | Rgs9 | Zfp940 |
| Alpk3 | Fbxo16 | Ly86 | Rmst | Zfp994 |
| Alx1 | Fsip1 | Lynx1 | Sarm1 | Zim3 |
| Amz1 | Galnt15 | Map3k13 | Scn5a | Zkscan7 |
| Angptl6 | Gbx1 | Marveld3 | Sf3a2 | Zpbp2 |
| Ankrd23 | Glrp1 | Mdga1 | Sgip1 |  |
| Ankrd55 | Gm10548 | Meig1 | Sgsm1 |  |
| Apol10b | Gm15413 | Mfsd7a | Slc16a14 |  |
| Arhgap8 | Gm15850 | Mlph | Slc16a3 |  |
| Arhgef37 | Gm16894 | Mogat1 | Slc24a2 |  |
| Bach2os | Gm17308 | Mucl3 | Slc25a23 |  |
| BC051019 | Gm26760 | Mx2 | Slc2a4 |  |
| Bicdl1 | Gm29688 | Myb | Slc2a6 |  |
| Brinp3 | Gm5464 | Mypn | Slc4a5 |  |
