# Supplementary material for: Laminin-α2 chain deficiency in skeletal muscle causes dysregulation of multiple cellular mechanisms
Source: Life Sci Alliance. 2024 Oct 8;7(12):e202402829. doi: 10.26508/lsa.202402829 (PMC11463332; doi:10.26508/lsa.202402829)
Supplement: Supplementary file 2 [file LSA-2024-02829_TableS2.docx]

**Supplementary Material**

**Supplementary Table 2.** List of genes obtained from the Venn diagram analysis comparing the differentially expressed genes (DEGs) (p-value 0.05, log2 fold change +/-1.5) of wildtype vs. *dy^W^* muscle fibers (in Figure 4) with gene ontology analysis using the GO:0030036 Actin Cytoskeleton Organization.

| Actin Cytoskeleton Organization | | | | | | | | | | | |
| --- | --- | --- | --- | --- | --- | --- | --- | --- | --- | --- | --- |
| Downregulated | | | | | | | | Upregulated | | | |
| Gene symbol | Log2 (FC) | Gene symbol | Log2 (FC) | Gene symbol | Log2 (FC) | Gene symbol | Log2 (FC) | Gene symbol | Log2 (FC) | Gene symbol | Log2 (FC) |
| Actg1 | -2,41 | Dner | -4,55 | Mylk3 | -8,97 | Smyd3 | -1,56 | Asf1a | 3,41 | Qki | 2,24 |
| Adgrb1 | -3,99 | Dock2 | -4,68 | Myorg | -4,02 | T | -7,30 | Bmpr2 | 2,09 | Rgs2 | 1,84 |
| Adra1b | -9,49 | Flt3l | -5,23 | Mypn | -2,41 | Tbx1 | -7,58 | Bnip2 | 1,85 | Tomm70a | 1,55 |
| Akap6 | -1,89 | G6pd2 | -7,43 | Naglu | -3,56 | Tbx5 | -7,49 | Ccn4 | 2,93 | Yy1 | 1,91 |
| Alpk2 | -4,82 | Gata4 | -8,34 | Neu2 | -4,32 | Tcf23 | -3,94 | Cfh | 3,40 | Zeb1 | 1,69 |
| Alpk3 | -5,02 | Gata6 | -4,51 | Nfatc2 | -2,88 | Tmem182 | -7,64 | Ctnnb1 | 1,82 |  |  |
| Ankrd23 | -6,06 | Hey2 | -7,86 | Nln | -2,84 | Tnfsf14 | -7,61 | Efnb2 | 2,26 |  |  |
| Asb2 | -5,66 | Isl1 | -7,99 | Nphs1 | -4,56 | Trim72 | -3,91 | Hes1 | 1,59 |  |  |
| B4galnt2 | -8,72 | Krt19 | -6,53 | Nrap | -9,72 | Ttn | -2,35 | Hnrnpu | 2,14 |  |  |
| Barx2 | -7,43 | Lama1 | -3,18 | P2rx2 | -6,80 | Wfikkn1 | -3,84 | Ifrd1 | 2,63 |  |  |
| Bhlha15 | -6,44 | Lmod1 | -7,52 | Pi16 | -4,38 | Wfikkn2 | -8,35 | Igf1 | 2,83 |  |  |
| Bmp10 | -7,30 | Lrrc27 | -3,03 | Pin1rt1 | -7,60 | Xirp1 | -7,98 | Igfbp5 | 3,03 |  |  |
| C3 | -3,94 | Msx1 | -6,15 | Pld3 | -4,12 | Xk | -4,26 | Lox | 3,47 |  |  |
| Cacna1s | -8,57 | Mybpc3 | -8,98 | Plpp7 | -8,14 | Zfp418 | -10,05 | Nr3c1 | 2,01 |  |  |
| Cd53 | -4,35 | Myh6 | -9,49 | Popdc2 | -5,65 |  |  | Pdgfra | 1,88 |  |  |
| Cntnap2 | -2,56 | Myh7 | -7,91 | Prdm6 | -7,47 |  |  | Ppp3ca | 2,33 |  |  |
| Cxcl9 | -7,74 | Myhas | -7,47 | Rpl3l | -7,49 |  |  | Ppp3cb | 2,06 |  |  |
