# Supplementary material for: Laminin-α2 chain deficiency in skeletal muscle causes dysregulation of multiple cellular mechanisms
Source: Life Sci Alliance. 2024 Oct 8;7(12):e202402829. doi: 10.26508/lsa.202402829 (PMC11463332; doi:10.26508/lsa.202402829)
Supplement: Supplementary file 3 [file LSA-2024-02829_TableS3.docx]

**Supplementary Material**

**Supplementary Table 3.** List of genes obtained from the Venn diagram analysis comparing the differentially expressed genes (DEGs) (p-value 0.05, log2 fold change +/-1.5) of wildtype vs. *dy^W^* muscle fibers (in Figure 4) with gene ontology analysis using the GO:0042692 Muscle Cell Differentiation

| Muscle Cell Differentiation | | | | | | | | | | | |
| --- | --- | --- | --- | --- | --- | --- | --- | --- | --- | --- | --- |
| Downregulated | | | | | | | | | | Upregulated | |
| Gene symbol | Log2 (FC) | Gene symbol | Log2 (FC) | Gene symbol | Log2 (FC) | Gene symbol | Log2 (FC) | Gene symbol | Log2 (FC) | Gene symbol | Log2 (FC) |
| Actb | -2,19 | Crhr2 | -8,26 | Insrr | -8,67 | Nlrp5 | -6,43 | Scin | -3,90 | Actr2 | 1,52 |
| Actg1 | -2,41 | Cryaa | -6,96 | Kash5 | -5,11 | Nphs1 | -4,56 | Serpinf2 | -5,49 | Antxr1 | 1,65 |
| Adgrb1 | -3,99 | Dmtn | -4,16 | Kbtbd13 | -5,22 | Nrap | -9,72 | Sh2b2 | -4,40 | Cald1 | 1,86 |
| Agap2 | -4,86 | Dnai3 | -3,99 | Krt19 | -6,53 | Ooep | -3,54 | Shank1 | -4,12 | Capza2 | 2,31 |
| Aif1l | -2,09 | Dock2 | -4,68 | Limk1 | -3,00 | Parvg | -8,61 | Shroom3 | -3,35 | Cul3 | 1,89 |
| Alox15 | -7,26 | Elmo3 | -2,87 | Lmod1 | -7,52 | Pawr | -8,91 | Specc1 | -2,12 | Epb41l2 | 1,74 |
| Ankrd23 | -6,06 | Ermn | -6,89 | Mlst8 | -5,28 | Pfn4 | -8,10 | Spire2 | -7,53 | Fmnl2 | 2,07 |
| Aqp2 | -8,17 | Espn | -8,74 | Msrb1 | -3,10 | Phactr3 | -7,69 | Sptb | -2,43 | Gja1 | 2,34 |
| Arhgap40 | -9,09 | Espnl | -8,29 | Mybpc3 | -8,98 | Pick1 | -1,95 | Sptbn5 | -8,23 | Marcks | 3,41 |
| Arhgap44 | -3,54 | Fam107a | -4,51 | Myh14 | -3,11 | Pls1 | -8,79 | Srcin1 | -3,99 | Myo1b | 1,67 |
| Asb2 | -5,66 | Fchsd1 | -5,58 | Myh6 | -9,49 | Ppargc1b | -4,46 | Tac1 | -8,33 | Nck1 | 2,55 |
| Bmp10 | -7,30 | Fhdc1 | -5,57 | Myh7 | -7,91 | Prkcq | -3,90 | Tacr1 | -8,30 | Nckap1 | 1,88 |
| Bst1 | -8,85 | Fmnl1 | -2,89 | Mylk3 | -8,97 | Pstpip2 | -4,89 | Trpm2 | -8,40 | Pdgfra | 1,88 |
| Capn10 | -3,01 | Frmd5 | -4,45 | Myo15a | -4,10 | Pxn | -3,94 | Ttn | -2,35 | Phactr2 | 2,37 |
| Carmil2 | -8,71 | Frmd7 | -6,79 | Myo1a | -8,11 | Rhoh | -10,38 | Ush1c | -6,05 | Pls3 | 2,60 |
| Carmil3 | -5,28 | Fzd10 | -9,16 | Myo3b | -7,00 | Rhpn1 | -9,34 | Vil1 | -3,57 | Rdx | 1,66 |
| Cdc42bpb | -2,55 | Ghrl | -5,94 | Myo5b | -4,63 | Rnd1 | -6,29 | Wasf3 | -2,81 | Rock2 | 1,58 |
| Cln3 | -2,47 | Ghsr | -7,51 | Myo5c | -7,00 | Rnd2 | -3,22 | Wipf3 | -5,80 | Sptbn1 | 1,93 |
| Clrn1 | -7,84 | Grhl3 | -7,82 | Myo7a | -2,35 | Rtkn | -3,55 | Xirp1 | -7,98 | Tgfbr1 | 1,95 |
| Coro7 | -2,84 | Hip1r | -4,92 | Myo7b | -4,23 | S1pr2 | -2,30 |  |  | Twf1 | 2,41 |
| Cpne6 | -9,17 | Hrg | -5,79 | Mypn | -2,41 | Samd14 | -3,42 |  |  | Zeb2 | 1,73 |
