# Supplementary material for: Laminin-α2 chain deficiency in skeletal muscle causes dysregulation of multiple cellular mechanisms
Source: Life Sci Alliance. 2024 Oct 8;7(12):e202402829. doi: 10.26508/lsa.202402829 (PMC11463332; doi:10.26508/lsa.202402829)
Supplement: Supplementary file 4 [file LSA-2024-02829_TableS4.docx]

**Supplementary Material**

**Supplementary Table 4.** List of genes obtained from the Venn diagram analysis comparing the differentially expressed genes (DEGs) (p-value 0.05, log2 fold change +/-1.5) of wildtype vs. *dy^W^* muscle fibers (in Figure 4) with gene ontology analysis using the GO:0007049 Cell Cycle.

| Cell Cycle | | | | | | | | | | | |
| --- | --- | --- | --- | --- | --- | --- | --- | --- | --- | --- | --- |
| Downregulated | | | | | | | | Upregulated | | | |
| Gene symbol | Log2 (FC) | Gene symbol | Log2 (FC) | Gene symbol | Log2 (FC) | Gene symbol | Log2 (FC) | Gene symbol | Log2 (FC) | Gene symbol | Log2 (FC) |
| 1700028K03Rik | -7,72 | E4f1 | -3,17 | Misp | -4,97 | Spata17 | -7,57 | Actr2 | 1,52 | Nipbl | 1,63 |
| 4933427D14Rik | -4,31 | Ecrg4 | -4,38 | Morc2b | -7,28 | Spdya | -7,87 | Adamts1 | 1,99 | Orc4 | 1,91 |
| Actb | -2,19 | Enkd1 | -4,07 | Mov10l1 | -4,89 | Spdye4a | -7,69 | Angel2 | 1,74 | Pcna | 2,18 |
| Actl6b | -8,90 | Espl1 | -2,89 | Mre11a | -2,95 | Spire2 | -7,53 | Apc | 1,83 | Pds5b | 2,37 |
| Actr5 | -3,00 | Exo1 | -3,23 | Mrgprb2 | -6,87 | Spo11 | -7,27 | Arid2 | 1,64 | Phf10 | 2,09 |
| Ajuba | -2,77 | Fam107a | -4,51 | Msx1 | -6,15 | Sstr5 | -8,10 | Atrx | 1,64 | Pkd2 | 2,14 |
| Alox8 | -6,04 | Fkbp6 | -7,72 | Myb | -3,78 | Stag3 | -2,87 | Bnip2 | 1,85 | Plk2 | 1,60 |
| Ankfn1 | -9,28 | Flt3l | -5,23 | Nlrp5 | -6,43 | Stk33 | -7,79 | Btg1 | 2,09 | Ppp1r12a | 1,84 |
| Ankrd31 | -3,41 | Fsd1 | -8,35 | Nr2e1 | -3,49 | Stox1 | -8,47 | Ccnb2 | 2,12 | Ppp2ca | 1,91 |
| Ankrd53 | -5,87 | Fzd9 | -8,06 | Nudt15 | -6,77 | Syce1l | -8,02 | Ccng2 | 2,01 | Ppp2cb | 2,31 |
| Apbb1 | -4,87 | Gata3 | -3,86 | Nuf2 | -2,25 | Sycp2l | -8,11 | Ccni | 1,60 | Ppp2r2d | 1,58 |
| Aurkc | -8,09 | Gata4 | -8,34 | Ooep | -3,54 | Taf6 | -1,99 | Ccnl1 | 2,01 | Ppp3ca | 2,33 |
| Bid | -4,59 | Gata6 | -4,51 | Ovol1 | -7,67 | Tas2r121 | -7,28 | Cdc123 | 2,15 | Prpf40a | 1,58 |
| Birc7 | -7,80 | Gen1 | -2,47 | Pard6b | -5,60 | Tbx1 | -7,58 | Cdc27 | 1,90 | Pum2 | 1,94 |
| Brca2 | -3,41 | Gjc2 | -7,46 | Parp9 | -3,29 | Tert | -8,45 | Cdc73 | 1,67 | Rad21 | 2,23 |
| Brinp2 | -4,94 | Gm20824 | -6,76 | Pax6 | -5,69 | Tesmin | -8,78 | Chmp5 | 1,89 | Rdx | 1,66 |
| Brinp3 | -4,56 | Gm4297 | -6,63 | Pcid2 | -4,04 | Tex12 | -7,83 | Ckap2 | 2,29 | Rgs2 | 1,84 |
| Brip1 | -2,44 | Gm5934 | -7,99 | Phgdh | -2,91 | Tex19.1 | -7,49 | Cltc | 1,88 | Rock2 | 1,58 |
| Brme1 | -8,05 | Gm773 | -7,44 | Piwil1 | -8,48 | Tex19.2 | -7,52 | Csnk1a1 | 1,60 | Rps6ka3 | 1,93 |
| Btbd18 | -9,99 | Gmnc | -7,72 | Piwil4 | -5,96 | Tex24 | -8,62 | Ctcf | 1,86 | Sbds | 1,72 |
| Btg1c | -7,01 | Gpr132 | -7,05 | Plk5 | -8,63 | Tgm1 | -4,89 | Ctnnb1 | 1,82 | Septin11 | 2,23 |
| Btn2a2 | -7,67 | Hepacam | -7,86 | Pml | -2,62 | Tjp3 | -3,24 | Cul3 | 1,89 | Skil | 3,10 |
| Camk2b | -3,00 | Hepacam2 | -5,87 | Prdm11 | -4,60 | Tm4sf5 | -5,41 | Dctn6 | 1,93 | Smarca5 | 1,52 |
| Ccnf | -2,37 | Hnf4a | -9,06 | Prdm9 | -3,35 | Tmem67 | -3,24 | Ddx3x | 2,82 | Smc2 | 1,94 |
| Cdk15 | -3,76 | Hyal1 | -4,57 | Prkcq | -3,90 | Trim36 | -4,35 | Dr1 | 1,58 | Smc3 | 1,79 |
| Cdk20 | -7,96 | Iho1 | -3,13 | Psma8 | -4,14 | Trim71 | -3,89 | Eid1 | 2,40 | Smc4 | 1,69 |
| Cdk5rap1 | -3,43 | Ing4 | -8,28 | Rab11fip3 | -2,29 | Trim75 | -7,47 | Eif4e | 1,92 | Son | 2,65 |
| Cenpo | -5,14 | Ins1 | -6,28 | Rad51c | -4,66 | Trp63 | -4,55 | Epb41l2 | 1,74 | Sptbn1 | 1,93 |
| Cenpt | -2,29 | Insm2 | -6,75 | Rcc1 | -1,59 | Trp73 | -3,95 | Fap | 2,46 | Stag2 | 1,72 |
| Clgn | -9,10 | Kash5 | -5,11 | Recql5 | -2,21 | Ttbk1 | -8,95 | Gja1 | 2,34 | Tardbp | 2,18 |
| Cntd1 | -7,09 | Kifc5b | -3,48 | Rgs14 | -9,14 | Tuba8 | -7,37 | Gnai3 | 2,08 | Tcim | 3,92 |
| Crnn | -6,18 | Klhdc8b | -4,97 | Rmi2 | -9,07 | Tubb4a | -8,45 | Gtf2b | 2,06 | Tlk2 | 1,85 |
| Crocc | -2,99 | L3mbtl1 | -4,79 | Rnf112 | -6,82 | Tube1 | -5,79 | Haus3 | 2,47 | Top2b | 1,87 |
| Ctc1 | -2,82 | Lep | -7,26 | Rnf8 | -2,78 | Tunar | -9,01 | Heca | 2,30 | Trim37 | 1,66 |
| Cts7 | -8,67 | Lif | -7,55 | Rsph1 | -3,81 | Ube2u | -4,00 | Hes1 | 1,59 | Tsc22d2 | 2,05 |
| Cul9 | -3,56 | Lig3 | -3,25 | Rtkn | -3,55 | Ush1c | -6,05 | Hnrnpu | 2,14 | Uchl5 | 2,51 |
| Cuzd1 | -7,36 | Lig4 | -5,17 | Rxfp3 | -8,32 | Usp26 | -7,96 | Igf1 | 2,83 | Ufl1 | 2,09 |
| Cyp27b1 | -6,83 | M1ap | -7,17 | Septin1 | -3,66 | Usp44 | -7,63 | Ik | 1,83 | Usp47 | 1,68 |
| D7Ertd443e | -9,74 | Madd | -3,81 | Sgo2b | -7,00 | Xrcc3 | -4,20 | Ing1 | 2,10 | Wapl | 1,71 |
| Ddx11 | -2,40 | Mael | -7,06 | Shcbp1l | -2,97 | Zbtb49 | -3,65 | Kif2a | 1,85 | Yy1 | 1,91 |
| Deup1 | -7,56 | Majin | -6,73 | Sipa1 | -2,41 | Zfp365 | -8,61 | Larp7 | 1,85 | Zfp207 | 1,54 |
| Dmrt1 | -5,93 | Map10 | -8,47 | Six3 | -4,77 | Zfp369 | -2,93 | Lrrcc1 | 2,25 | Zfp36l2 | 3,01 |
| Dmrtc2 | -6,65 | Map1s | -1,88 | Slc25a31 | -5,69 |  |  | Mbtd1 | 1,68 |  |  |
| Dnmt3l | -4,06 | Mapk15 | -8,51 | Slc26a8 | -3,70 |  |  | Mki67 | 1,56 |  |  |
| Dpf1 | -4,06 | Mdc1 | -2,11 | Slc6a4 | -5,82 |  |  | Nabp1 | 1,86 |  |  |
| E2f1 | -3,10 | Mei1 | -10,02 | Slfn1 | -7,61 |  |  | Nfia | 1,73 |  |  |
| E2f2 | -4,02 | Meig1 | -7,67 | Spag8 | -5,94 |  |  | Nfib | 2,10 |  |  |
