# Supplementary material for: Laminin-α2 chain deficiency in skeletal muscle causes dysregulation of multiple cellular mechanisms
Source: Life Sci Alliance. 2024 Oct 8;7(12):e202402829. doi: 10.26508/lsa.202402829 (PMC11463332; doi:10.26508/lsa.202402829)
Supplement: Supplementary file 5 [file LSA-2024-02829_TableS5.docx]

**Supplementary Material**

**Supplementary Table 5.** List of genes obtained from the Venn diagram analysis comparing the differentially expressed genes (DEGs) (p-value 0.05, log2 fold change +/-1.5) of wildtype vs. *dy^W^* muscle fibers (in Figure 4) with gene ontology analysis using the GO:0006281 DNA Repair.

| DNA repair | | | | | | | | | |
| --- | --- | --- | --- | --- | --- | --- | --- | --- | --- |
| Downregulated | | | | | | Upregulated | | | |
| Gene symbol | Log2 (FC) | Gene symbol | Log2 (FC) | Gene symbol | Log2 (FC) | Gene symbol | Log2 (FC) | Gene symbol | Log2 (FC) |
| Actb | -2,19 | Hrob | -3,69 | Poln | -3,14 | Actr2 | 1,52 | Smc3 | 1,79 |
| Actl6b | -8,90 | Kash5 | -5,11 | Prdm9 | -3,35 | Arid2 | 1,64 | Smc4 | 1,69 |
| Actr5 | -3,00 | Kdm4d | -8,68 | Pwwp3a | -2,24 | Asf1a | 3,41 | Supt20 | 1,67 |
| Adprs | -2,62 | Lig3 | -3,25 | Rad51c | -4,66 | Atrx | 1,64 | Uchl5 | 2,51 |
| Apbb1 | -4,87 | Lig4 | -5,17 | Recql5 | -2,21 | Bod1l | 1,71 | Ufl1 | 2,09 |
| Ascc1 | -3,80 | Majin | -6,73 | Rmi2 | -9,07 | Eny2 | 2,09 | Usp47 | 1,68 |
| Brca2 | -3,41 | Mdc1 | -2,11 | Rnf8 | -2,78 | Gtf2h2 | 1,57 | Usp7 | 2,11 |
| Brip1 | -2,44 | Mgme1 | -3,11 | Spire2 | -7,53 | Kin | 2,48 | Xrn2 | 1,98 |
| Brme1 | -8,05 | Mgmt | -4,67 | Spo11 | -7,27 | Mbtd1 | 1,68 | Yy1 | 1,91 |
| Cyren | -3,86 | Morc2b | -7,28 | Supt7l | -3,30 | Nabp1 | 1,86 |  |  |
| Ddx11 | -2,40 | Mre11a | -2,95 | Taf6 | -1,99 | Nipbl | 1,63 |  |  |
| Dpf1 | -3,41 | Neil1 | -4,88 | Tdg-ps | -5,09 | Pcna | 2,18 |  |  |
| Endov | -6,17 | Neil2 | -9,34 | Tex12 | -7,83 | Pds5b | 2,37 |  |  |
| Exo1 | -3,23 | Ooep | -3,54 | Ttf2 | -3,54 | Phf10 | 2,09 |  |  |
| Eya2 | -8,60 | Parp9 | -3,29 | Ung | -4,01 | Psme4 | 2,00 |  |  |
| Fan1 | -9,80 | Pml | -2,62 | Xrcc3 | -4,20 | Rad21 | 2,23 |  |  |
| Gen1 | -2,47 | Pold2 | -1,91 | Zfp365 | -8,61 | Smarca5 | 1,52 |  |  |
| Ggn | -7,87 | Polh | -3,65 |  |  | Smc2 | 1,94 |  |  |
