# Supplementary material for: Laminin-α2 chain deficiency in skeletal muscle causes dysregulation of multiple cellular mechanisms
Source: Life Sci Alliance. 2024 Oct 8;7(12):e202402829. doi: 10.26508/lsa.202402829 (PMC11463332; doi:10.26508/lsa.202402829)
Supplement: Supplementary file 6 [file LSA-2024-02829_TableS6.docx]

**Supplementary Material**

**Supplementary Table 6.** List of genes obtained from the Venn diagram analysis comparing the differentially expressed genes (DEGs) (p-value 0.05, log2 fold change +/-1.5) of wildtype vs. *dy^W^* muscle fibers (in Figure 4) with gene ontology analysis using the GO: 0006979 Response to Oxidative Stress.

| Oxidative Stress Response | | | | | | | | | |
| --- | --- | --- | --- | --- | --- | --- | --- | --- | --- |
| Downregulated | | | | | | | | Upregulated | |
| Gene symbol | Log2 (FC) | Gene symbol | Log2 (FC) | Gene symbol | Log2 (FC) | Gene symbol | Log2 (FC) | Gene symbol | Log2 (FC) |
| Adipoq | -7,22 | Keap1 | -2,50 | Nqo1 | -4,05 | Slc4a1 | -5,43 | Hsph1 | 1,92 |
| Adprs | -2,62 | Lck | -2,98 | Nudt15 | -6,77 | Slc4a11 | -9,40 | Idh1 | 1,82 |
| Aldh3b1 | -8,20 | Lcn2 | -7,74 | Pawr | -8,91 | Sod3 | -4,29 | Pdgfra | 1,88 |
| Alox5 | -4,55 | Lpo | -6,76 | Pax2 | -7,14 | Stc2 | -5,98 | Ppp2cb | 2,31 |
| Cryaa | -6,96 | Hnf1a | -8,99 | Pex10 | -4,97 | Stox1 | -8,47 | Prdx3 | 1,82 |
| Epor | -6,10 | Meak7 | -5,49 | Pml | -2,62 | Tacr1 | -8,30 | Psip1 | 2,09 |
| Epx | -7,41 | Mmp3 | -3,31 | Ppargc1b | -4,46 | Tat | -6,90 | Sod1 | 2,46 |
| G6pd2 | -7,43 | Mpv17l | -6,58 | Ptprn | -9,08 | Thg1l | -5,23 |  |  |
| Gpx2 | -8,50 | Mtf1 | -3,05 | Pyroxd1 | -3,90 | Tlr4 | -6,58 |  |  |
| Gpx5 | -8,16 | Naglu | -3,56 | Rbm11 | -7,73 | Trpm2 | -8,40 |  |  |
| Gpx6 | -7,68 | Neil1 | -4,88 | Reg3b | -7,57 | Ucp3 | -8,08 |  |  |
| Hk3 | -4,05 | Ngb | -7,51 | Rgs14 | -9,14 | Wnt16 | -5,51 |  |  |
| Hyal1 | -4,57 | Nme8 | -8,41 | Rnf112 | -6,82 |  |  |  |  |
