# Supplementary material for: Laminin-α2 chain deficiency in skeletal muscle causes dysregulation of multiple cellular mechanisms
Source: Life Sci Alliance. 2024 Oct 8;7(12):e202402829. doi: 10.26508/lsa.202402829 (PMC11463332; doi:10.26508/lsa.202402829)
Supplement: Supplementary file 7 [file LSA-2024-02829_TableS7.docx]

**Supplementary Material**

**Supplementary Table 7.** List of genes obtained from the Venn diagram analysis comparing the differentially expressed genes (DEGs) (p-value 0.05, log2 fold change +/-1.5) of wildtype vs. *dy^W^* muscle fibers (in Figure 4) with gene ontology analysis using the GO:0007005 Mitochondria Organization.

| Mitochondrion Organization | | | | | | | | | | | |
| --- | --- | --- | --- | --- | --- | --- | --- | --- | --- | --- | --- |
| Downregulated | | | | | | | | Upregulated | | | |
| Gene symbol | Log2 (FC) | Gene symbol | Log2 (FC) | Gene symbol | Log2 (FC) | Gene symbol | Log2 (FC) | Gene symbol | Log2 (FC) | Gene symbol | Log2 (FC) |
| 2610042  L04Rik | -5,37 | Dmac2 | -5,09 | Mgme1 | -3,11 | Slc25a33 | -2,25 | Atg3 | 2,09 | Ssbp1 | 2,42 |
| Adck1 | -1,89 | Dnaja3 | -2,10 | Miga2 | -3,55 | Slc4a5 | -8,61 | Cfh | 3,40 | Timm22 | 2,18 |
| Afg1l | -4,36 | Dnajc11 | -2,19 | Mpv17l | -6,58 | Spata18 | -7,14 | Dctn6 | 1,93 | Tomm20 | 1,91 |
| Afg3l1 | -1,78 | Eya2 | -8,60 | Myh14 | -3,11 | Stox1 | -8,47 | Hsp90aa1 | 2,30 | Tomm70a | 1,55 |
| Agtpbp1 | -1,79 | Ggnbp1 | -6,42 | Nod2 | -6,10 | Stpg1 | -7,94 | Igf1 | 2,83 | Tug1 | 1,79 |
| Atcay | -5,65 | Hap1 | -9,01 | Nptx1 | -4,00 | Tert | -8,45 | Man2a1 | 2,29 | Vps54 | 1,97 |
| Atg4d | -8,10 | Hip1r | -4,92 | P2rx7 | -4,70 | Thg1l | -5,23 | Marcks | 3,41 | Yme1l1 | 1,62 |
| Atg9b | -8,88 | Hrk | -7,16 | Pisd | -2,10 | Tnfsf10 | -5,36 | Mcl1 | 1,98 | Zdhhc6 | 2,11 |
| Bhlha15 | -6,44 | Irgm2 | -7,97 | Pla2g6 | -4,20 | Tomm40 | -2,41 | Ogt | 2,34 |  |  |
| Bid | -4,59 | Kif28 | -7,83 | Ptpn5 | -9,38 | Trp73 | -3,95 | Ppp2cb | 2,31 |  |  |
| Capn10 | -3,01 | Letm1 | -2,45 | Rcc1l | -3,27 | Tymp | -7,90 | Prdx3 | 1,82 |  |  |
| Cntnap2 | -2,56 | Lig3 | -3,25 | Sco1 | -4,50 |  |  | Pum2 | 1,94 |  |  |
| Cryaa | -6,96 | Lncbate1 | -5,92 | Siah3 | -6,41 |  |  | Ralbp1 | 1,89 |  |  |
| Dhodh | -3,60 | Lyrm7 | -5,00 | Slc25a31 | -5,69 |  |  | Snx7 | 1,81 |  |  |
